# Straining at a Gnat

**Published:** 1885-05

**Authors:** Chas. E. Francis

**Affiliations:** New York


### "STRAINING AT A GNAT!"

BY DR. CHAS. E. FRANCIS, NEW YORK.

A paper was recently read before the Central Illinois Dental Society, and published in the March number of the *Ohio State Journal*, on "The Poisonous Effects of Amalgam Fillings."

The author assumes that "the poisonous effects are due to evaporation of mercury from the fillings, which is going on at all times under ordinary temperatures." Also, that "mercurial poisoning manifests itself variously—sometimes immediately upon inserting the filling, and again, not for years." "The symptoms in the first instance," he observes, "are characterized by a metallic taste and peculiar sensation, similar to that experienced in holding both poles of a galvanic battery ; and particularly is this the case where the amalgam is in close contact with other fillings, or when touched with a silver fork." And further, that similar results are also produced in partaking of "some kinds of food." According to the views of the essayist, "a shock is experienced," and this "shock" is manifestly a liliputian shower of mercurial poison !

In proof of this "theory," a case is cited where, in Philadelphia, some tempting but treacherous "raw oysters" were incautiously introduced within the buccal cavity of a too confiding individual, wherein was "a large amalgam filling which had been recently inserted, producing a shock followed by pain so intense that the filling had to be removed."

Mercy on us ! What *does* such sort of testimony amount to ? Similar "shocks" and "pains" are of frequent occurrence where no amalgam is present. With tin and gold fillings such experiences have been related and *re*-related over and over again, for scores of years back, as every dentist must know. A "raw oyster" coming in contact with one of our own incisors at a commencement supper in this same city of "Brotherly-love," gave a shock that produced unutterable agony; and that filling also "had to be removed." In this case, however, the tooth contained a "recently inserted" *gold* filling, and there was no amalgam whatever in the mouth. Perhaps the luscious bivalves of the Delaware Bay region partake largely of the nature of *electric eels*, and resent with a stinging farewell rebuke the indignity of being served in a nude and vivisected condition on the half-shell.

We have also on various occasions experienced thermal shocks in other teeth filled with the "precious metal," when eating fruits or taking into the mouth food or drink of unsuitable temperature, and have felt galvanic shocks when silver, tin or other metals have come in contact with the gold. Why then should not similar shocks occur where *amalgam* is employed ?

The average amalgam filling is larger than that of gold. The larger the filling, the greater the danger to be apprehended from sudden thermal changes—the prime cause of pulp irritation and pulpitis. Large gold or tin foil fillings are quite as likely to convey thermal or galvanic shocks to pulps nearly exposed as fillings of amalgam ; and such results have proved quite too common in the practice of those who utterly ignore plastic stoppings. As a rule, it is unsafe to allow metal fillings of any sort to be placed in close proximity with living pulps, excepting, perhaps, some of the metallic oxides.

We are further informed that "the most common pathological condition as a result of mercurial poisoning is *ptyalism*." The normal flow of saliva is given as "three pounds per twenty-four hours," and if the flow exceeds this amount we have an "abnormal condition." Our Chicago friend also states that, among his patients, those having several amalgam fillings, or both gold and amalgam fillings, "are almost always afflicted with ptyalism!"

It is reasonable to presume that human nature is pretty much the same in the different sections of our country, especially in *functional* peculiarities, yet we occasionally hear strange and wonderful stories concerning Chicago folks—especially the "girls"—and this may be among their peculiarities. To state *precisely* the normal flow of saliva hereabouts, would be preposterous. We have *all* conditions of saliva-flowing "in these parts," and crackers are often eaten for a wager!

While some mouths might remind one of a summer drought, others appear more like an autumnal deluge. Fillings of amalgam, with or without the gold, seem to make precious little difference in regulating this flow, and the closest observations of years fail to reveal such marked influences. And in regard to the conditions of the gums, amalgam has just about as little to do in this respect. The worst appearing cases of diseased gums that have come within the scope of our observation, have been in mouths where no amalgam was found. *Imagination* will conjure up many strange notions, and strange notions are wonderfully prevalent in these days. As yet, neither galvanic nor thermal shocks have been universally labelled "poison," and even though it is possible that constant galvanic action may favor decalcification of enamel rods, its influence on the general system has not been considered *baneful*. Galvanic doctors, galvanic combs, brushes, belts, bracelets, rings, plasters, baths, etc., are advertised as "*aids* to health," hence we observe that imagination runs blindly in either direction.

In considering the decrease in weight of an amalgam filling by the evaporation of mercury, which (it is stated) is going on little by little for years, we think it requires the biggest sort of fancied conjecture to perceive any marked effect exhibited on the general health, and it is a question of very grave doubt if any such evaporation takes place, or any perceptible diminution in a filling can be demonstrated. So far as *experiments* are concerned, to offset this "gaseous" theory, we can refer to experiments of a most elaborate nature made some years ago by Dr. E. A. Bogue, of New York, the late Prof. Hitchcock, of Boston, and others, who, sparing neither time, labor nor expense, for many months devoted themselves to the task of experimenting with amalgam in order to ascertain if any perceptible loss could take place in stoppings of this material when inserted in the teeth. The fact was clearly demonstrated, that the degree of heat requisite to produce mercurial vapor could not possibly be tolerated in the mouth of a living individual; also, that no solvent of amalgam pervades the precincts of the oral cavity.

Reports of these experiments were published in the *Dental Cosmos*, as transactions of the *N. Y. Odontological Society* at its December session for 1874.

Some individuals, overstocked with effeminate credulity, suffer constant fear, lest in some mysterious manner they may get poisoned. With them, every real or fancied ailment is attributed to either "malaria" or "mercury." These two formidable "bugbears" are brought out in turn, and each covers heaps of ignorance. The probabilities are that not once in ten times are they the *real* cause of mischief laid at their doors.

Where peridentitis or alveolar abscess proceeds from the root of a tooth in which there is an amalgam filling, it is too frequently diagnosed "mercurial poisoning." When, however, the same conditions appear where the stopping is of gold, the trouble is dignified by the term "neuralgia." Either diagnosis is, of course, equally senseless.

A New York physician of the Hahnemann type, one who boasts of a large practice, once informed us that at an early period of his life a "*villainous* country doctor" gave him a dose of calomel, and as a result his teeth had been "full of mercury ever since." A careful inspection of the mouth gave no evidence of mercurial mischief. A pulpless superior molar, containing a large gold filling, had at times given him slight annoyance, but all the other teeth and gums were in a remarkably healthy condition. The calomel notion was exceedingly "vapory."

Another physician of the same school, in his vain endeavors to benefit a juvenile patient by administering ineffective "high-dilution" agents, declared that the "potency" of his "*remedies*" was counteracted by the presence of amalgam fillings in the child's teeth, and that the child was suffering from "mercurial poisoning." Subsequent examination by the dentist who had caused the "mischief," disclosed the fact that the child's teeth contained five stoppings of white gutta percha, and one of tin foil. All were in excellent condition, and no amalgam had ever been used. The feelings of the almost frantic mother were appeased, and the idiotic dispenser of pellets was advised to shoulder his own foolery, instead of falsely charging his ill-success to the operations of the dentist, whose reputation with this family he would have thus jeopardized.

A prominent and much esteemed dentist of New York treated and filled a devitalized superior molar with a stopping of oxy-chlor. of zinc. As the root-canals were closed too soon, peridentitis followed, and the cheek became much swollen. The dentist was sharply denounced for introducing a filling of a poisonous nature; "metallic poisoning" was the diagnosis of the leather-brained attendant, and another dentist was visited, who did his best to correct the erroneous impression, and to lull the storm of indignation which the operation and its effects had caused.

Cases similar to the above are by no means uncommon, and sensible dentists, instead of encouraging such ridiculous notions, should strive to dissipate them. Many individuals defer visits to the dentist, until driven to seek relief from relentless attacks of odontalgia. As a rule, such people desire to retain their teeth, and expect their dentist not only to relieve the immediate pain, but to so treat them that no after trouble is possible. Indeed, they presume that after such miserable offenders are once "fixed," they are better than ever before, and thoroughly insured from further annoyance for a lifetime! If the pain recurs, instead of blaming themselves for their unpardonable neglect, they are sure to make a scape-goat of the faithful dentist, who did all in his power to benefit them.

Our Chicago brother closed his paper with this trite paragraph:

"The profession and the public will welcome a filling which shall take the place of amalgam." To this we heartily respond, "amen."

We candidly confess, that although cases occur where amalgam is our last resort, we never employ it without a degree of reluctance, and for the simple reason that it tends to slightly bulge from cavity margins. This does not always happen, as is proved by the exhibit in many instances where it has remained perfect and unchanged for years, yet, where such a tendancy is *possible,* it lessens confidence. So far as injury to the system is concerned by "mercurial poisoning," we believe this to be *sheer nonsense.* As soon would we believe that all the fishes that sport in the pellucid waters of Lake Superior would become distressed should we cast therein a grain of calomel, or a spoonful of salt.

The majority of our ablest physicians consider mercury the "anchor of safety" in many cases which they are called upon to treat, and prescribe it day after day in doses of from two to fifteen grains, whereas, all the possible waste in an amalgam filling through a lifetime from "evaporation" would be so exceedingly minute that only the most exalted imagination could conceive it.

In accounting for many of the "ills that human flesh is heir to," the imagination sometimes gets terribly strained in gigantic efforts to "swallow a gnat," while "camels" are engorged with little ceremony. Wasting one's efforts over such vapory, visionary conjectures, is too much like grasping at the shadow of a shadow, or a futile searching for "the infinitesimal abstraction of ethereal inanity."

Verily—"a much ado about nothing!"

